# Current Evidence and Insights about Genetics in Thoracic Aorta Disease

**DOI:** 10.1155/2013/962097

**Published:** 2013-12-24

**Authors:** Gianluigi Bisleri, Lorenzo Bagozzi, Claudio Muneretto

**Affiliations:** Division of Cardiac Surgery, University of Brescia Medical School, Cardiochirurgia SSVD, Spedali Civili, Piazzale Spedali Civili 1, 25123 Brescia, Italy

## Abstract

Thoracic aortic aneurysms have been historically considered to be caused by etiologic factors similar to those implied in abdominal aortic aneurysms. However, during the past decade, there has been increasing evidence that almost 20% of thoracic aortic aneurysms may be associated with a genetic disease, often within a syndromic or familial disorder. Moreover, the presence of congenital anomalies, such as bicuspid aortic valve, may have a unique common genetic underlying cause. Finally, also sporadic forms have been found to be potentially associated with genetic disorders, as highlighted by the analysis of rare variants and expression of specific microRNAs. We therefore sought to perform a comprehensive review of the role of genetic causes in the development of thoracic aortic aneurysms, by analyzing in detail the current evidence of genetic alterations in syndromes such as Marfan, Loeys-Dietz, and Ehler-Danlos, familial or sporadic forms, or forms associated with bicuspid aortic valve.

## 1. Introduction

Aneurysm is defined as a permanent localized dilatation of an artery, having at least a 50% increase in diameter compared to the expected normal diameter of the artery itself [1]. The most common sites of aortic aneurysms are abdominal being caused in the majority of instances by atherosclerosis [2].

Conversely, the etiology of the thoracic aortic aneurysm (TAA) remains not clearly defined in most instances and likely to be multifactorial; in fact, risk factors such as atherosclerosis, history of smoking, chronic obstructive pulmonary disease, hypertension, gender, advanced age, and high body mass index seem to yield less relevance than genetic predisposition in TAA [3].

Recently, there has been increasing evidence in the literature that more than 20% of TAA may have predisposing genetic factors, in particular in association with familial aggregation [4, 5]. These findings have driven an intense research aimed at a better definition of the genetic and molecular mechanisms involved in the pathogenesis of TAA; while syndromic disorders have been extensively studied with respect to clinical features and genetic background, also familial (nonsyndromic) forms have recently been demonstrated to be linked with single gene defects [6].

Moreover, there is increasing evidence that other aortic disorders with potential genetic etiology, such as the bicuspid aortic valve, could be correlated to a precocious onset of TAA, caused by a unique common genetic predisposition [7].

Finally, recent studies demonstrated that molecular mechanisms are different in genetic and nongenetic forms, first of all in the expression of metalloproteinases [8] and microRNAs [9].

The purpose of current review is therefore to analyze the causes leading to the development of TAA, in particular with a comprehensive overview of the genetic and molecular features of the different forms, such as Marfan, Loeys-Dietz, and Ehler-Danlos syndromes, familial (nonsyndromic) forms, TAA associated with bicuspid aortic valve, and finally sporadic forms.

## 2. Marfan Syndrome

In 1896, Antoine-Bernard Marfan, first described this syndrome following a clinical evaluation of a 5-years-old female patient [10]; only in 1931, the dominant autosomic feature of the syndrome could be defined [11].

The Ghent criteria, first reported in 1996 and reviewed in 2010 by Loeys et al., are currently utilized to diagnose this syndrome [12].

In the majority of instances, the Marfan syndrome is caused by a mutation of the FBN-1 gene, which codifies the fibrillin-1, a large 350 kDa glycoprotein that is sited in the extracellular matrix and is fundamental to maintain the integrity of the vessel wall, promoting the anchorage of smooth muscle cells (SMCs) to the matrix of elastin and collagen [13]. Recently it was also shown that fibrillin-1 interacts with TGF-beta signalling and, when FBN-1 is mutated, TGF-beta bioavailability and activity are increased [14].

Mutations in the FBN-1 identified to date are more than 2,000, distributed among the 65 gene exons (http://www.umd.be); such mutations are usually the same within a family but not across different familiar groups.

A second locus for Marfan syndrome, termed MFS2, was identified to be caused by mutations in TGFBRII and the phenotype may overlap with Loeys-Dietz syndrome [15].

## 3. Loeys-Dietz Syndrome

Loeys-Dietz syndrome was first reported in 2005 and phenotypically overlaps with Marfan syndrome and vascular Ehlers-Danlos syndrome, therefore leading to misdiagnosis among the above-mentioned syndromes [16].

This syndrome is associated with dilatation and dissection potentially affecting any arterial site; moreover, it can be associated with arterial tortuosity, thin and translucent skin with visible veins, exuberant scars, and craniofacial deformities, including bifid uvula and hypertelorism [17]. Recently, the Loeys-Dietz syndrome has been divided into two subtypes: LDS1 (also named “facial dysmorphogenic type” for notable cleft palate, craniosynostosis, and micrognathia) and LDS2 (or “vascular EDS-like type,” in which findings as visceral rupture, easy bruising, atrophic scars, joint laxity, and translucent skin are common).

The syndrome is caused by mutations affecting the genes for receptors 1 and 2 of transforming growth factor (TGFBR1 and TGFBR2); mutations of TGFBR2 are more frequent [18]. The mutations of the receptor for TGF-beta cause a paradoxical activation of the SMAD pathway, via its phosphorylation [19]. The mutations in the TGFBR2 occur in variable phenotypes according to the penetrance of the disease; it is therefore advisable to conduct a family screening based on molecular biology.

If LDS is suspected based on the clinical features, genetic testing for TGBR1 and TGFBR2 mutations should be performed since in such instances a patient may be more likely to suffer from aortic rupture and dissection at a younger age and even with smaller aortic diameter [18].

Finally, it has been recently reported that arterial tortuosity is a negative prognostic factor in patients with TGFBR2 mutations as well as in patients with familial forms of TAA from other etiologies [20].

## 4. Ehlers-Danlos Syndrome

The Ehlers-Danlos syndrome was identified for the first time in 1901 [21] and described as a dominant autosomal disease in 1949 [22]. The syndrome is classified in 11 subtypes, where the vascular variant is type IV (ESD-IV or vEDS) [23].

The mutations leading to this disease affect type 3 pro-collagen (COL3A1), which is the subtype of collagen most represented in the extracellular matrix of the vessels and organ walls [24].

The clinical features of vEDS are thin skin with visible veins, easy bruising, and characteristic facial features as thin pinched nose, thin lips, prominent ears, hollow cheeks, and tightness of skin over the face. Individuals with vEDS have significantly shortened life spans (50% mortality rate by the age of 48) due to the spontaneous rupture of visceral organs and blood vessels [24].

Scientific research on knockout Col3a1 mice and surgical data suggests that the vascular tissue is extremely weak and potentially cumbersome to deal with during aortic replacement. Therefore, elective surgery should be timely planned before aortic dissection could occur [25].

## 5. Familial (Nonsyndromic) Thoracic Aortic Aneurysms (FTAAs)

Despite the role of genetics having been widely described in patients with MFS, its impact also in familiar (nonsyndromic) forms of TAA has been elucidated only in the past few years.

Patients with a positive family history for TAA show clinical evidence of the disease at a significantly younger age than sporadic cases [4], albeit they do not express the features of aforementioned syndromes; hence, much interest has been manifested for FTAAs in the last decade. Among the genes most frequently responsible for FTAAs, ACTA2, MYH11, and SMAD3 are of utmost importance.

ACTA2 mutations are the most common cause of familial TAA identified to date, being accounting for nearly 15% of the disease [1].

The differentiated SMC expresses a peculiar repertoire of actins; a mutation of ACTA2 gene causes the absence of ACTA2-containing filaments in SMCs and an increased pool of unpolymerized ACTA2 [26]. Analysis of the aortic tissue also showed an increased percentage of proteoglycan, fragmentation and loss of elastic fibres, and a decreased rate of SMCs (typical findings of cystic medionecrosis); moreover, the SMCs are randomly orientated resembling the myocyte disarray typically found in hypertrophic cardiomyopathy.

Testing for ACTA2 is a class IIa recommendation in the most recent guidelines for patients with a thoracic aortic aneurysm and a family history of TAA [1].

Mutations in MYH11 can be found in less than 2% of the FTAA population; when such mutations are present, ascending aortic aneurysm and patent ductus arteriosus are often found in conjunction [27].

MYH11 encodes the smooth muscle myosin heavy chain (SM-MHC), a major specific contractile protein produced in SMC. The mutations observed in MHY11 may affect the assembly of myosin thick filaments. The mutations inhibit the SMC capacity to migrate, proliferate, and differentiate, thus hampering the closure of the ductus arteriosus and meanwhile leading to the development of an aneurysm.

Mutations in the gene SMAD3 are the most recent described in TAA. These mutations lead to clinical features similar to LDS albeit more likely to be associated with bone anomalies; in fact, in such instances the disease has been defined as aneurysm and osteoarthritis syndrome (AOS), which involves about 2% of TAA [28]. Because of its syndromic character, these mutations should be best classified in syndromic forms of TAA.

SMAD3 encodes for a key regulator in the TGF-beta pathway that activates or represses gene transcription. In particular, missense mutations substitute essential amino acids for trimer formation with SMAD3 and SMAD4, which is crucial for the translocation of the SMAD complex to the nucleus and for genes regulation. Connective tissue growth factor (CTGF) showed a markedly increased cytoplasmic expression in the medial vascular SMCs, to stimulate the production of collagen III (increased in media).

The studies made on the vascular tissues involved by SMAD3 mutation show a disarray of the medial layer with fragmentation and loss of elastic fibres, as well as cystic medial degeneration.

With respect to the skeletal features, a progressive loss of articular cartilage has been detected along with formation of large osteophytes, as typically observed in human osteoarthritis [28].

## 6. Bicuspid Aortic Valve and Thoracic Aortic Aneurysms

Ascending aortic dilatation occurs more frequently and in a younger age in patients with bicuspid aortic valves (BAV) than in patients with normal, trileaflets aortic valves (TAV). BAV is the most common congenital cardiac abnormality occurring in 0,46% to 1,37% of the population [29].

The aortic valve and ascending aorta share a common embryonic origin, because they both develop from neural crest cells. In the past, the altered pattern of blood flow caused by the BAV was believed to cause a mechanical stress against the wall of the ascending aorta, thus contributing to the development of an aneurysm. However, several BAV studies demonstrated, at a histological level, an increased rate of apoptosis of SMCs, also with the wall layers of a normal aorta [30].

In addition, histological analysis of such aortic walls provided further insights about the differences between a BAV and a TAV aorta; despite a similar wall thickness, the distance among the elastic lamellae is increased in the presence of BAV and the lamellae themselves have reduced with and a higher degree of fragmentation. These findings are always present in moderate dilatation with BAV, while in patients with TAV, they are statistically significant only when the aorta is severely dilated [31]; thus, such peculiar finding could be interpreted as an early marker of damage on the vessel walls.

The role of fibronectin has also been investigated; in fact, there are two variants of fibronectin, arising from alternative splicing of the same gene, which can be found also in the plasma in dimeric form and lacks the alternatively spliced extra domain A (EDA) and extra domain B (EDB). The exact functions of EDA and EDB in the vessel are not yet known, but they appear to be important for cell migration and proliferation and to have a critical role in vascular morphogenesis during embryogenesis. The differences in expression of EDA and EDB between BAV and TAV patients could support the hypothesis of an early mechanism of molecular damage. In this regard, Paloschi et al. demonstrated a significant reduction in the expression of fibronectin and its different splice forms in cells from BAV patients compared with those from TAV patients [32].

Among the genetic alterations leading to the development of BAV, some mutations have been identified in the NOTCH1 gene, which seem to cause also a higher degree of calcium deposition at the valvular level. More recently, a possible etiologic role has been demonstrated due to mutations of SIRT1, which in turn affects a decreased expression of the NOTCH signalling [33].

Molecular biology techniques have shown an altered expression of metalloproteinases and in particular an increase of gelatinases (MMP2) that degrade collagen type 4 and partially also the elastin and the fibrillar collagen [8]. This pattern of MMP has been recently associated with the expression of particular types of microRNA; the study conducted by Jones et al. through a regression model has demonstrated an inverse relationship between concentration of microRNA-29a and MMP2, thus inferring that some RNAs could modulate the transcription of certain metalloproteinases [9]. Additional studies have shown a pathogenetic role of MMP12 activity within the aortic wall; MMP12 is also recognized as macrophage-elastase; however, during histological analysis, it was not possible to demonstrate the presence of inflammatory infiltration [34].

## 7. Sporadic Thoracic Aortic Aneurysms (STAAs)

Sporadic TAAs do not show any familial aggregation or transmission; the precise cellular and molecular mechanisms remain poorly understood. They can originate in the setting of autoimmune diseases (such as giant cell arteritis, Takayasu's arteritis, rheumatoid arthritis, or Reiter's syndrome) and other disorders as well as traumatic conditions, albeit representing the minority of cases. In most instances, sporadic TAAs occur as degenerative conditions in association with age, hypertension, and smoking, despite having paucity of data regarding the precise pathophysiology.

Sporadic cases of TAA, not related to defined mutations, are associated with increased risk of TAA in first-degree relatives; it is usually reported that in 20% of apparently sporadic forms of TAA, another family member presents with TAA, indicating that some familial aggregation may exist [4].

Lemaire and colleagues explained that the 15q21.1 single nucleotide polymorphisms (SNPs), lying in a linkage-disequilibrium region containing the entire FBN1 gene, are associated with STAA [35]. This means that these variants in FBN1 gene alone could lead to the development of a thoracic aortic aneurysm, without a *“true”* mutation of the gene itself and the typical clinical features of Marfan Syndrome. In fact, previous studies demonstrated that FBN1 mutations can predispose individuals to thoracic aneurysms in the absence of MFS [36]; therefore, it is not surprising that the possible functional variant in FBN1 predisposes people to thoracic aortic disease in the absence of skeletal and ocular features of MFS.

A recent study by Prakash and colleagues demonstrated the role of rare copy number variants (CNVs) in genes regulation. In particular, TAA patients harbour a remarkable number of rare CNVs that are enriched for genes known to be involved in TAA or other vascular disorders. This team identified a total of 84 rare CNVs; since 13% of STAA analyzed patients harbour these rare CNVs, it seems that such CNVs may contribute to a greater proportion of TAA cases than single gene mutations [37].

The expression of tissutal microRNAs (miRNAs) has been the focus of recent studies. The miRNAs are small molecules of endogenous noncoding RNA, single strand, of 20–22 nucleotides that are part of a large network of regulatory genes and perform different functions, being the posttranscriptional regulation best described to date. In the study of Liao et al. a statistically significant difference was shown in the overexpression of 18 miRNAs and subexpression of 56 miRNA in patients with thoracic aortic dissection [38]. In particular, the role of some miRNA families (as 29 and 30) seems to be crucial: miRNAs 29a and 29c are involved in the control of the deposition of collagen types I and III and their underexpression may enhance the deposition; miRNAs 143 and 145 are involved in the modulation and the differentiation of SMCs, and finally miRNA 30 appears to be involved in the pathway of MAP-kinases.

Jones et al. described the relation between the miRNAs pattern and tissue expression of MMPs, establishing the evidence that some miRNAs may modulate the transcription of certain MMPs by pairing the corresponding RNA-messenger [9].

## 8. Conclusions

During the past decades there has been an improved understanding of the role of genetic alterations in presence of thoracic aortic disease.

Syndromic forms (such as Marfan, Ehlers-Danlos, and Loeys-Dietz) should always be confirmed by means of genetic testing; familiar (nonsyndromic) forms should similarly have patients screened at least for ACTA-2 mutations, as per guidelines recommendations [1].

In the presence of bicuspid aortic valve, the demonstration of genetic alterations at the level of NOTCH-1 and SIRT-1 could suggest a strict monitoring of patients due to the higher likelihood to develop thoracic aortic aneurysm.

Finally, the paucity of data regarding genetic mutations in patients with sporadic forms of thoracic aortic disease does not provide enough evidence to support systematic genetic screening to date, albeit it is foreseeable that the technical improvements in genome sequencing along with the reduction of procedural costs could allow for a wider use of genetic screening also in this subset of patients in the near future.
